# Fractal characteristics of overburden fissures in shallow thick coal seam mining in loess gully areas

**DOI:** 10.1371/journal.pone.0274209

**Published:** 2022-09-27

**Authors:** Jianwei Li, Xinwei Guo, Xiangye Wu, Shijiang Chen, Ningbo Zhang

**Affiliations:** 1 College of Mining and Coal, Inner Mongolia University of Science and Technology, Baotou, China; 2 School of Mines, China University of Mining & Technology, Xuzhou, China; China University of Mining and Technology, CHINA

## Abstract

The problems of water scarcity and ecological fragility are common in the loess gully area. To research the distribution and evolution of the overburden fissures and quantitatively analyze them have certain theoretical and engineering significance for realizing the evaluation of overburden damage degree and safe and green mining. This paper takes the 6102 working face of Chuancao Gedan Coal Mine as the engineering background. The development law and distribution characteristics of overburden fissures caused by the mining of shallow coal seams in the loess gully area were studied by the combination of physical similarity simulation, numerical similarity simulation and fractal theory. The results show that the fractal dimension change of the overburden fissures caused by the shallow mining of coal seam groups in the loess gully area can be divided into three stages during the mining process of the working face. Repeated mining causes the activation and development of overburden fissures, the fractal dimension increases significantly, and the regularity of changes weakens. The magnitude of the stress near the working face and the fluctuation times of the stress in the goaf have an influence on the change of the fractal dimension of the overburden fissures. According to the development angle and the fractal dimension of the overburden fissures, the overburden rock above the goaf is divided into the collapse fissure area, the compaction fissure area, and the vertical fissure area. Overburden fissures develop violently in the vertical fissure area, the overburden fissures in the compaction fissure area are mostly transverse fissures, and the overburden fissures in the caving fissure area are irregular.

## 1. Introduction

Most coal resources in loess gully areas of Western China are characterized by shallow burial, a large mining thickness, and suitable for mechanized mining [1]. Coal seam mining causes overburden instability, movement, and deformation and produces a large number of mining fissures, which not only cause ecological disasters, such as groundwater loss, cutting-induced surface damage, and vegetation withering in the mining area, but also make some overburden fissures connect the surface with the underground goaf and working face, resulting in safety accidents, such as air leakage, water and sand eruption in the working face [2, 3]. Therefore, it is helpful for the green and safe mining of coal resources to study the development law of the overburden fissures affected by coal seam mining.

Scholars usually use a variety of methods to study the development law of fissures in the overburden rock of the goaf. For example, Cheng et al. [4] studied the distribution of mining-induced fractures in an overburden with the help of a high precision microseismic monitoring system. Deng [5], Zhang [6], and Zhang et al. [7] studied the fracture development law in the process of coal seam advancement by numerical simulations. Tan et al. [8] studied the failure evolution of an overburden caused by multi-coal seam mining based on field measurements, using a similar material model.

In the study of overburden fissures, many scholars use fractal theory to quantitatively analyze them. As a very active theory, fractal theory was first proposed by Mandelbrot in 1975. Its basic idea is to describe and study objective things from the perspective of fractional dimensions and mathematical methods, which is closer to the description of the real attributes and states of complex systems [9]. In recent years, fractal theory has achieved fruitful results in the study of overburden fissures. For example, Scholz [10], Walsh [11], and Jackson et al. [12] studied the fractal characteristics of fault number size distribution, fault displacement distribution, and fault spacing distribution. Xie [13] summarized the mathematical basis and methods of fractal application and established the relationship between macroscopic mechanical quantities and geometric physical quantities of rock mass materials by using fractal theory, which laid the foundation for related research. Yu et al. [14] used fractal theory to quantitatively described the spatial distribution and evolution of the fracture network in the mining area. Wang [15], Miao [16], and Liu et al. [17] studied the fracture evolution process and failure mode of mining rock masses with initial fractures and revealed the fractal dimension evolution law of mining rock mass fractures. In underground resource mining, the development law of overburden fissures is affected by different factors [18–20]. Song [21], Zhang [22], and Cheng et al. [23] studied the stress fracture distribution and evolution characteristics of surrounding rock under the influence of dual mining and discussed the relationship between the fractal dimension and mining stress. Pan [24] and Zhang et al. [25] explored the development law of overburden fractures after mining double thick coal seams by using a combination of similarity simulation and numerical simulation and analyzed the fractures by using fractal dimension. Yang [26] established a coal seam permeability coefficient calculation model based on the fractal dimension of mining fractures, and revealed and determined the gas migration law and fracture development law in the coalbed methane-enriched area. Wang [27] and Hu et al. [28] studied the gas transport properties of fractured shale gas reservoirs, and a new multiscale fractal transport model with an effective porosity model was proposed based on the fractal theory and the multilayer fractal Frenkel-Halsey-Hill (FHH) adsorption.

The above scholars have done a lot of research on the development law of overburden fissures under the leveled surface, but few scholars have considered the development law of overburden fissures under the gully surface. Therefore, based on previous research, this paper considers the influence of special topographical conditions in the loess gully area on the instability movement of overlying rock, and studies the development law of overburden fissures in the mining of shallow coal seam groups. The quantitative analysis of overburden fissures is carried out by using fractal theory. The research results provide a certain reference value for the study of overburden fissures development law in loess gully area.

## 2. Engineering background

**Fig 1:**
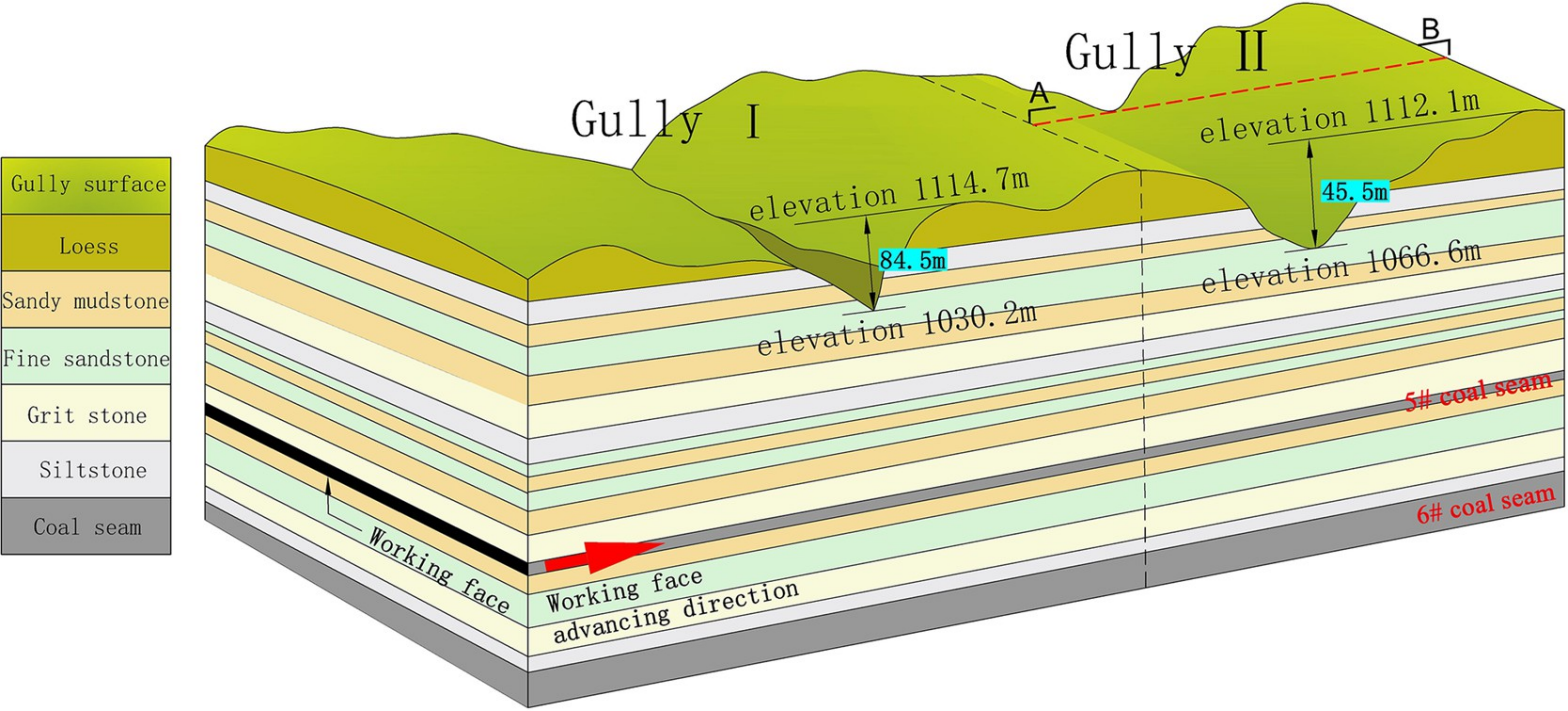
Schematic diagram of the occurrence state of loess gullies on the surface.

**Fig 2:**
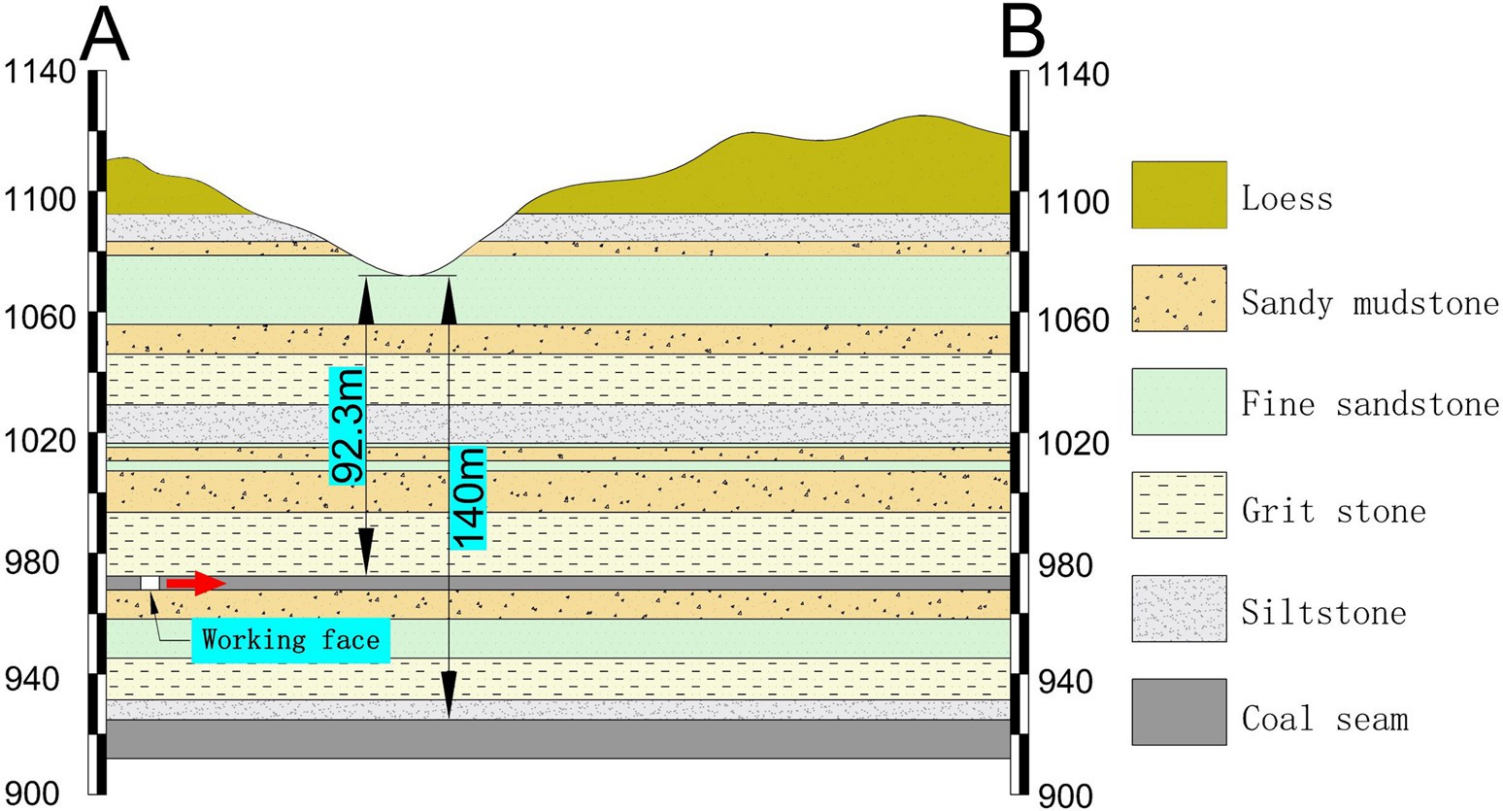
Schematic diagram of the AB section of the Gully II.

Chuancao Gedan Coal Mine is located in the south of the Jungar Coalfield in Inner Mongolia. The area is a typical erosive loess plateau with large topographic fluctuations and vertical and horizontal gullies. There are two mineable coal seams in the overlying rock in the area, namely 5# coal and 6# coal. There are two deep ravines on the surface of the area, namely Gully I and Gully II. The highest point of Gully I is 1114.7 m, the lowest point is 1030.2 m, and the maximum level variation is 84.5 m. The highest point of Gully II is 1112.1 m, the lowest point is 1066.6 m, and the maximum level variation is 45.5 m. The advancing direction of the working face is vertical to the gully trend. The occurrence state of the loess gullies on the surface is shown in Fig 1. Gully II was selected as the study area and the AB section is shown in Fig 2.

## 3. Development law of overburden fissures in goaf in gully area

### 3.1. Construction of a physical similarity simulation model

Based on the occurrence of overlying strata of Chuancao Gedan coal mine, and considering the similarity ratio between the actual engineering size and the physical model. A model of simulation experiment of physical similarity was established [29].

**Fig 3:**
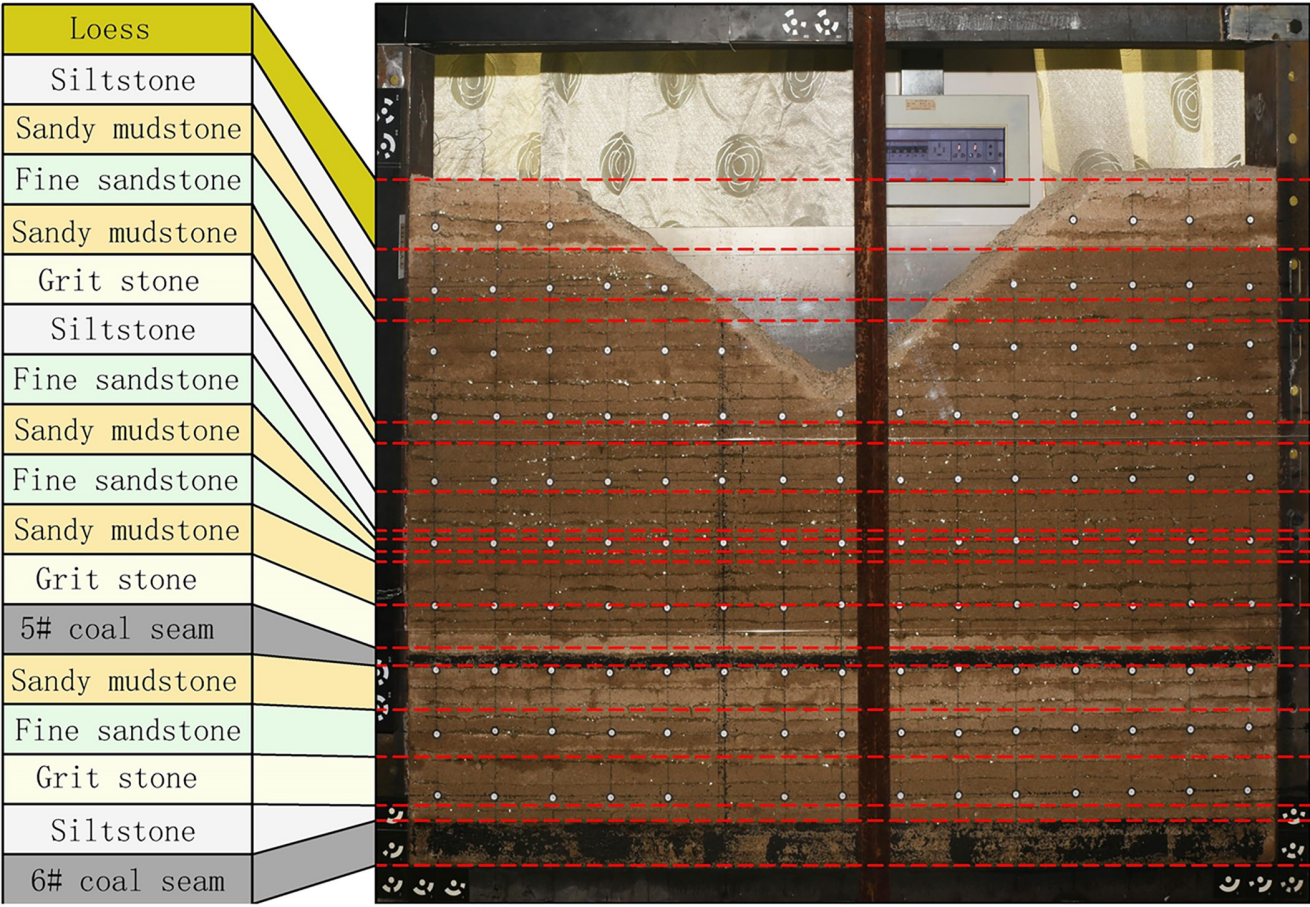
Physical similar simulation model.

The dimensions of the physical similarity simulation test bench are: length × width × height = 1500 mm × 160 mm × 1500 mm. According to similarity theory, the geometric similarity ratio was 1:200, the bulk density similarity constant was 1.67, the stress similarity constant was 334 and the time similarity constant was 14.14. In the physical similarity simulation experiment, the simulation materials for each rock stratum were made of fine river sand, calcium carbonate, gypsum, and water according to a certain proportion number. They are laid in layers along the horizontal direction, and mica powder is spread between the layers to simulate the weak surface of the rock stratum. In the process of model-making, the compressive strength was taken as the main similarity condition and meets the similarity criterion. Table 1 shows similar model parameters and ratios. Fig 3 shows a physical similar simulation model.

**Table 1:** Similar model parameters and ratios.

Serial number	Lithology	Thickness(cm)	Proportion number	Sand quality (kg)	Calcium carbonate quality (kg)	Gypsum quality (kg)	Water consumption (L)
1	Loess	8.9	873	27.0	2.4	1.0	3.0
2	Siltstone	4.6	455	12.4	1.5	1.5	1.5
3	Sandy mudstone	2.3	673	7.4	0.9	0.4	0.9
4	Fine sandstone	11.4	573	32.2	4.5	1.9	3.9
5	Sandy mudstone	5	673	16.0	1.9	0.8	1.9
6	Grit stone	8.4	555	25.1	2.5	2.5	3.0
7	Siltstone	6.4	455	17.2	2.2	2.2	2.2
8	Fine sandstone	0.7	573	2.0	0.3	0.1	0.2
9	Sandy mudstone	2.2	673	7.0	0.8	0.4	0.8
10	Fine sandstone	1.7	573	4.8	0.7	0.3	0.6
11	Sandy mudstone	6.9	673	22.0	2.6	1.1	2.6
12	Grit stone	10.6	555	31.7	3.2	3.2	3.8
13	5# coal seam	2.3	773	7.2	0.7	0.3	0.8
14	Sandy mudstone	4.8	673	15.3	1.8	0.8	1.8
15	Fine sandstone	6.5	573	18.4	2.6	1.1	2.2
16	Grit stone	6.9	555	20.6	2	2.1	2.5
17	Siltstone	3.3	455	8.9	1.1	1.1	1.1
18	6# coal seam	6.5	773	20.4	0.6	0.3	0.7

In the model excavation process, leave 10 cm on each side of the model to protect the coal pillars. First, excavate the 5# coal seam, the mining height of the 5# coal seam is 2.3cm, and the step distance of each excavation is 5 cm, and the mining length is 110 cm. Then excavate the 6# coal seam, the mining height of the 6# coal seam is 6.5 cm, the excavation step is 5 cm, and the mining length is 110 cm. After each excavation, photographs were taken to record the fracture morphology of the overlying rock.

### 3.2. Development law of overburden fissures

**Fig 4:**
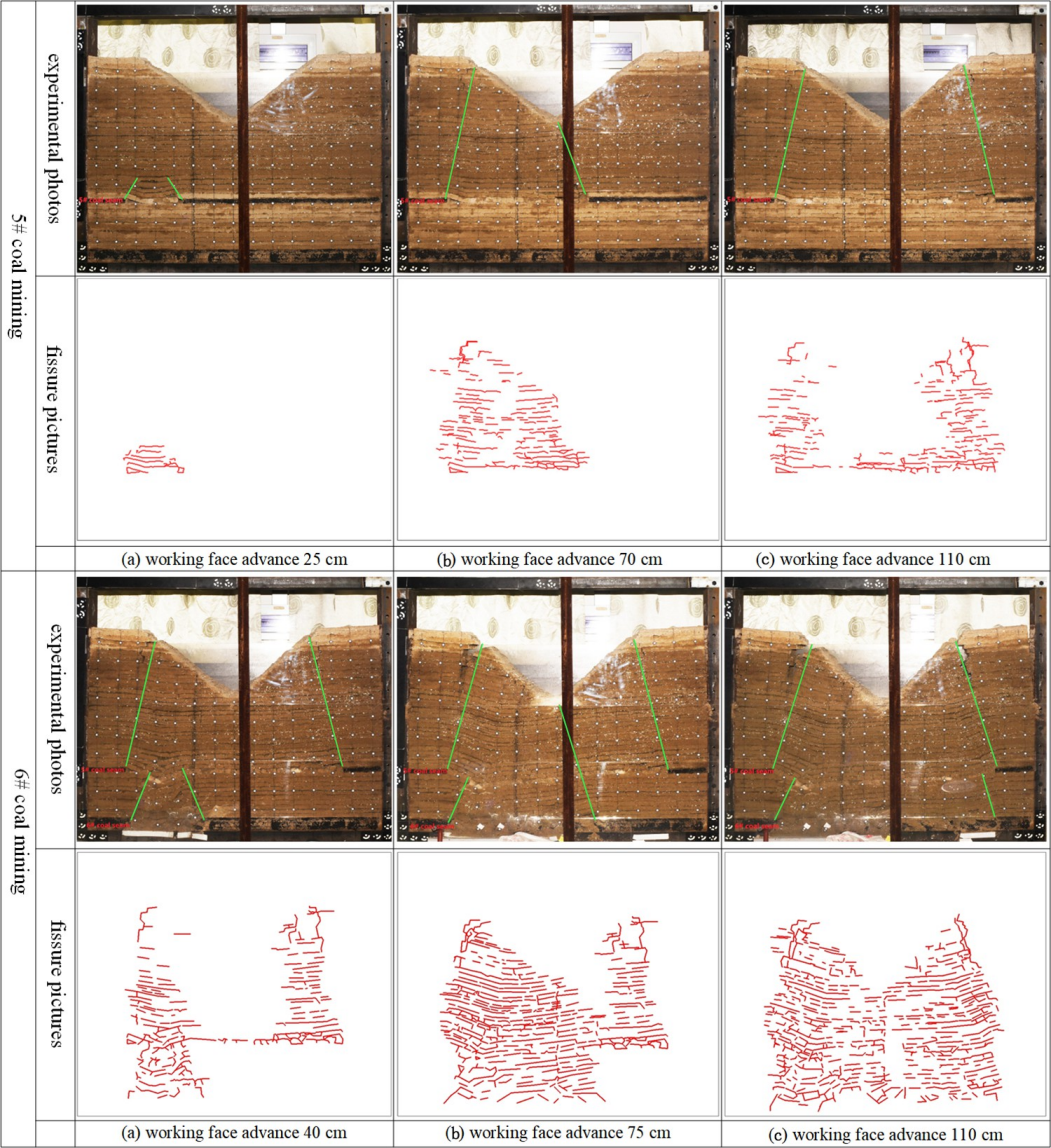
Experimental photos and fissures distribution.

According to the photos shown in Fig 4, the development law of overburden fissures in coal seam group mining is analyzed.

#### 3.2.1. 5# coal seam mining

When the working face advances to 20 cm. The direct roof first collapses, and fissures appeared in the damaged rock formation.

When the working face advances to 70 cm. The movement of the overlying rock instability affects the surface, the surface of the left slope in the gully area has obvious subsidence, a large number of fissures appear in the damaged rock formation, and longitudinal fissures appear on the surface.

The mining was stopped when the working face advanced to 110 cm. The instability movement of the overlying rock affected the right slope of the gully area, and the surface of the right slope appeared to show obvious subsidence. The fissures in the overlying rock at the gully bottom tend to be closed, the longitudinal fissures on the surface of the gully bottom tend to be closed, and there are obvious longitudinal fissures in the overlying rock on both slopes of the gully.

#### 3.2.2. 6# coal seam mining

When the working face advances to 40 cm. The rock layers between the 6# coal seam and the 5# coal seam are damaged. The instability movement of overlying rock affects the 5# coal seam goaf. In the 5# coal seam goaf, the overlying rock has secondary instability movement, and the fissures in the overlying rock increase sharply.

When the working face advances to 75 cm. The movement of the overlying rock instability affects the surface, the surface of the left slope in the gully area has secondary subsidence, the closed longitudinal fissures on the surface at the gully bottom reopened, the longitudinal fissures on both sides of the gully slope are further expanded and deeper.

The mining was stopped when the working face advanced to 110 cm. Affected by the secondary instability movement of the overlying rock, the overburden rock fissures further increased in the overall goaf, and the longitudinal fissures also increased in the surface.

### 3.3. Quantitative analysis of overburden fissures

Based on the results of the above physical similarity simulation experiment, using the box dimension calculation method in fractal theory, the fractal dimension of the overburden fissures is calculated [30]. The basic calculation principle of the box dimension method is to use a square box with side length *r* to cover the fissures, and the size of the box can be changed. Assuming that the side length of a given box is *r*, the total number of boxes *N*(*r*) required to cover the fissures can be calculated, and the fractal dimension *D* can be calculated by Formula (1). The calculation formula for the box dimension is as follows:

D=−limr→0logN(r)log1r (1)

Where, *D* is the fractal dimension; *N*(*r*) is the number of boxes covering the fissures; *r* is the side length of the box.

**Fig 5:**
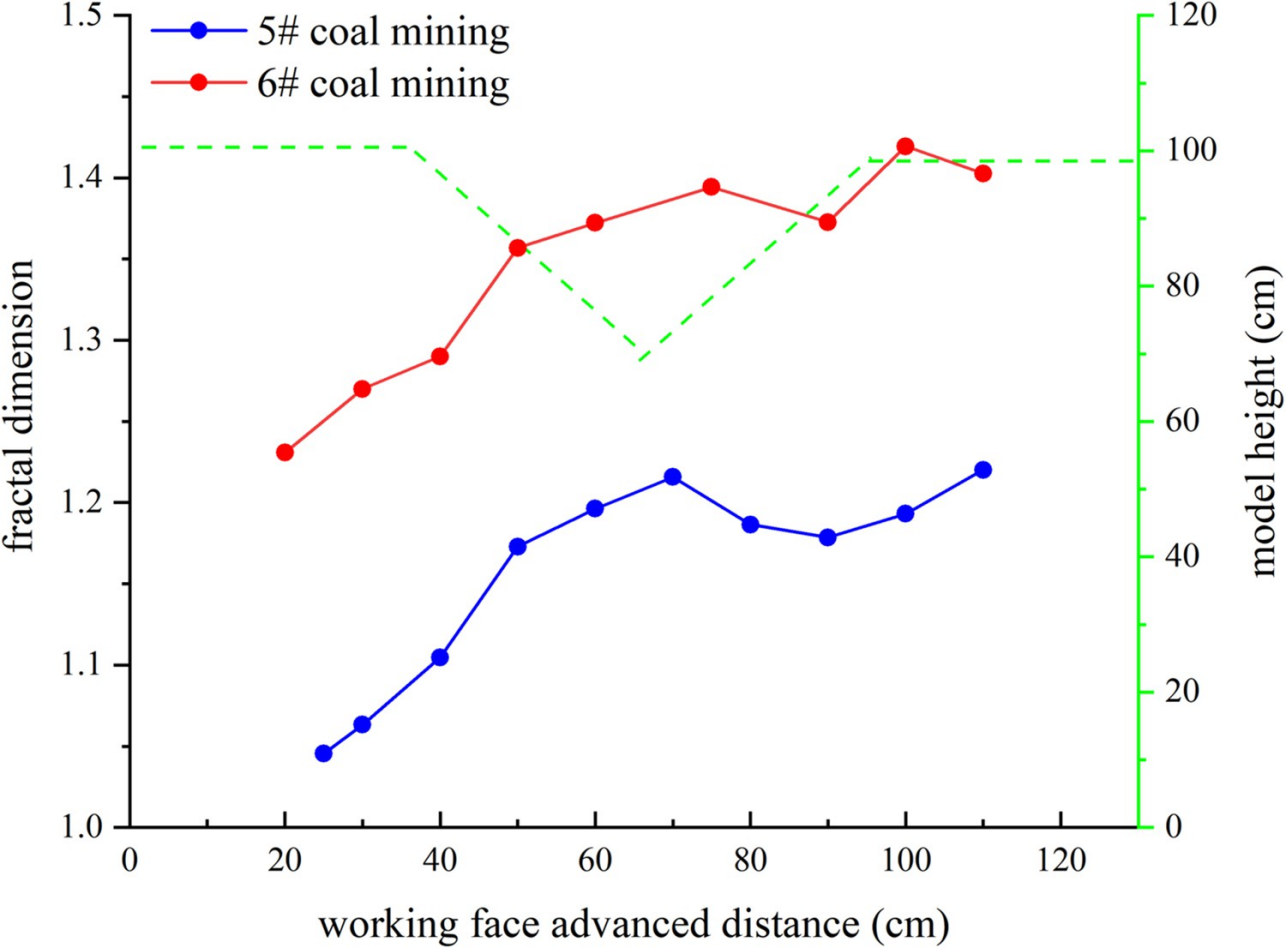
Line chart of fractal dimension of overburden fissures.

The fractal dimension and correlation coefficient calculation results of overburden fissures under different advancing distances of the working face are shown in Table 2. A line graph is drawn according to the fractal dimension of the overburden fissures, as shown in Fig 5.

**Table 2:** Fractal dimension of overburden fissures.

5# coal seam mining		6# coal seam mining	
Advancing distance (m)	Fractal dimension	Advancing distance (m)	Fractal dimension
50	1.0454	40	1.2308
60	1.0632	60	1.2699
80	1.1047	80	1.2901
100	1.1729	100	1.3568
120	1.1963	120	1.3722
140	1.2157	150	1.3943
160	1.1865	180	1.3726
180	1.1786	200	1.4194
200	1.1931	220	1.4026
220	1.2202		

As can be seen in Fig 5, the development degree of the overburden fissures is positively correlated with its fractal dimension. The fractal dimension of the overburden fissure changes in stages, and the change rule is as follows:

#### 3.3.1. 5# Coal seam mining

*3*.*3*.*1*.*1*. *Fractal dimension increase stage of mining under the left slope (The excavation distance of the working face is 0–70 cm)*. The working face is below the left slope of the gully, the thickness and weight of the overlying rock gradually decreases as the working face advances. The fissures generated in the overlying rock on the working face are gradually reduced and the possibility of the fissures being compacted by the weight of the overlying rock is small. Due to the damaged rock blocks on the goaf tend to move to the center of the gully, and new fissures are created in the overlying rock on the goaf, and the fissures in the overlying rock on the working face are compacted. When the working face under the left slope is in the mining stage, the fissures formation rate is faster than the compaction rate, the number of fissures in the overlying rock gradually increases, and the fractal dimension increases. When the working face is advanced to 70cm, the overlying rock on the left slope is completely destroyed and subsides. There are the most fissures in the overlying rock in the goaf, and the fractal dimension of the overburden fissures reaches the maximum at this stage.

*3*.*3*.*1*.*2*. *Fractal dimension decrease stage of mining under the right slope (The excavation distance of the working face is 70–90 cm)*. The working face is below the right slope of the gully, the thickness and weight of the overlying rock gradually increases as the working face advances. The fissures generated in the overlying rock on the working face are gradually increase and the possibility of the fissures being compacted by the weight of the overlying rock is large. Fewer fissures are created in the overlying rock as the left slope overburden is no longer moving. When the working face is in this stage of mining, the fissures formation rate is slower than the compaction rate, and the fractal dimension of the overlying fissures decreases.

*3*.*3*.*1*.*3*. *Fractal dimension increase stage of mining under the right slope (The excavation distance of the working face is 90–110 cm)*. The working face is below the right slope of the gully, the thickness and weight of the overlying rock further increases as the working face advances. Numerous fissures appeared in the overburden near the working face. And the damaged rock blocks in the slope on the right side of the gully also tends to move to the center of the gully, more fissures are generated in the overlying rock. When the working face is in this stage of mining, the fissures formation rate is faster than the compaction rate, and the fractal dimension increases.

In the process of mining 6# coal seam, the fractal dimension change law of overburden fissures is similar to that of 5# coal mining, but the inflection point of fractal dimension broken line is different from 5# coal mining.

The mining of the 5# coal seam has caused most of the overlying rock layers to be destroyed and the formation of fissures. Mining the 6# coal seam makes the broken rock blocks move violently and the fissures further increase, making the fractal dimension larger. Because the 6# coal seam is buried deeper than the 5# coal seam, the 6# coal seam needs to be mined for a longer distance to affect the bottom of the surface gully. When the 6# coal seam is mined to about 75cm, the overburden fissures generated by the mining of the working face affect the bottom of the surface gully, and the fractal dimension is the largest at this stage. The inflection point of the change in fractal dimension of the overburden fissures during the 6# coal mining lags behind that of the 5# coal mining.

## 4. Influence of mining stress on development law of overburden fissures

### 4.1. Construction of numerical simulation model

Using the 3DEC numerical simulation software, according to the actual size of the project in engineering background, the numerical simulation model of shallow-buried coal seam mining in the loess gully area was established. The size of the model is: length × wide × height = 340 m × 5 m × 172 m. The model is calculated using the Mohr-Coulomb yield criterion, and the stress-displacement mixed boundary. The upper surface of the model is the ground surface, no additional stress is applied, and the horizontal compressive stress that changes with the depth is applied to the side. The mechanical parameters of the model are shown in Tables 3 and 4.

**Table 3:** Mechanical parameters of coal and rock block.

Lithology	Density (kg·m^-3^)	Bulk modulus (GPa)	Shear modulus (GPa)	Cohesive (MPa)	Friction (°)	Tensile strength (MPa)
Loess	2375	0.05	0.08	0.8	8	0.01
Siltstone	2440	1.70	8.1	1.8	29	2.40
Sandy mudstone	2536	1.79	6.3	10.0	31	1.32
Fine sandstone	2241	1.59	4.5	8.4	34	2.05
Sandy mudstone	2536	1.79	6.3	10.0	31	1.32
Grit stone	2405	2.37	5.6	7.8	35	2.20
Siltstone	2440	1.70	3.7	1.8	29	2.40
Fine sandstone	2241	1.59	4.5	8.4	34	2.05
Sandy mudstone	2536	1.79	6.3	10.0	31	1.32
Fine sandstone	2241	1.59	4.5	8.4	34	2.05
Sandy mudstone	2536	1.79	6.3	10.0	31	1.32
Grit stone	2405	2.37	5.6	7.8	35	2.20
5# coal seam	1430	1.30	1.3	1.0	24	2.05
Sandy mudstone	2536	1.79	6.3	10.0	31	1.32
Fine sandstone	2241	1.59	4.5	8.4	34	2.05
Grit stone	2405	2.37	5.6	7.8	35	2.20
Siltstone	2440	1.70	3.7	1.8	29	2.40
6# coal seam	1430	1.30	1.3	1.0	24	2.05
Siltstone	2440	1.70	3.7	1.8	29	2.40

**Table 4:** Mechanical parameters of the coal and rock joint plane.

Lithology	Normal stiffness (GPa)	Tangential stiffness (GPa)	Cohesive (MPa)	Friction (°)	Tensile Strength (MPa)
Loess	0.10	0.10	0.05	12.0	0.06
Siltstone	6.90	6.90	1.50	27.2	1.80
Sandy mudstone	6.10	6.10	0.40	12.0	0.48
Fine sandstone	1.90	1.90	2.00	15.0	2.00
Sandy mudstone	6.10	6.10	0.40	12.0	0.48
Grit stone	0.40	0.40	1.60	25.6	2.16
Siltstone	14.90	14.89	1.63	27.2	1.80
Fine sandstone	1.90	1.90	2.00	35.0	2.20
Sandy mudstone	6.10	6.10	0.40	15.0	0.40
Fine sandstone	0.09	0.08	0.60	10.0	0.60
Sandy mudstone	6.10	6.10	0.36	15.0	0.36
Grit stone	0.40	0.40	1.44	32.0	1.62
5# coal seam	8.33	8.33	0.40	15.0	0.30
Sandy mudstone	6.10	6.10	0.40	15.0	0.40
Fine sandstone	18.48	18.48	0.80	20.0	0.80
Grit stone	0.30	0.30	1.50	31.0	1.20
Siltstone	0.0052	0.0052	0.20	17.0	0.17
6# coal seam	8.33	8.33	0.40	15.0	0.30
Siltstone	1.898	1.90	2.00	35.0	2.20

**Fig 6:**
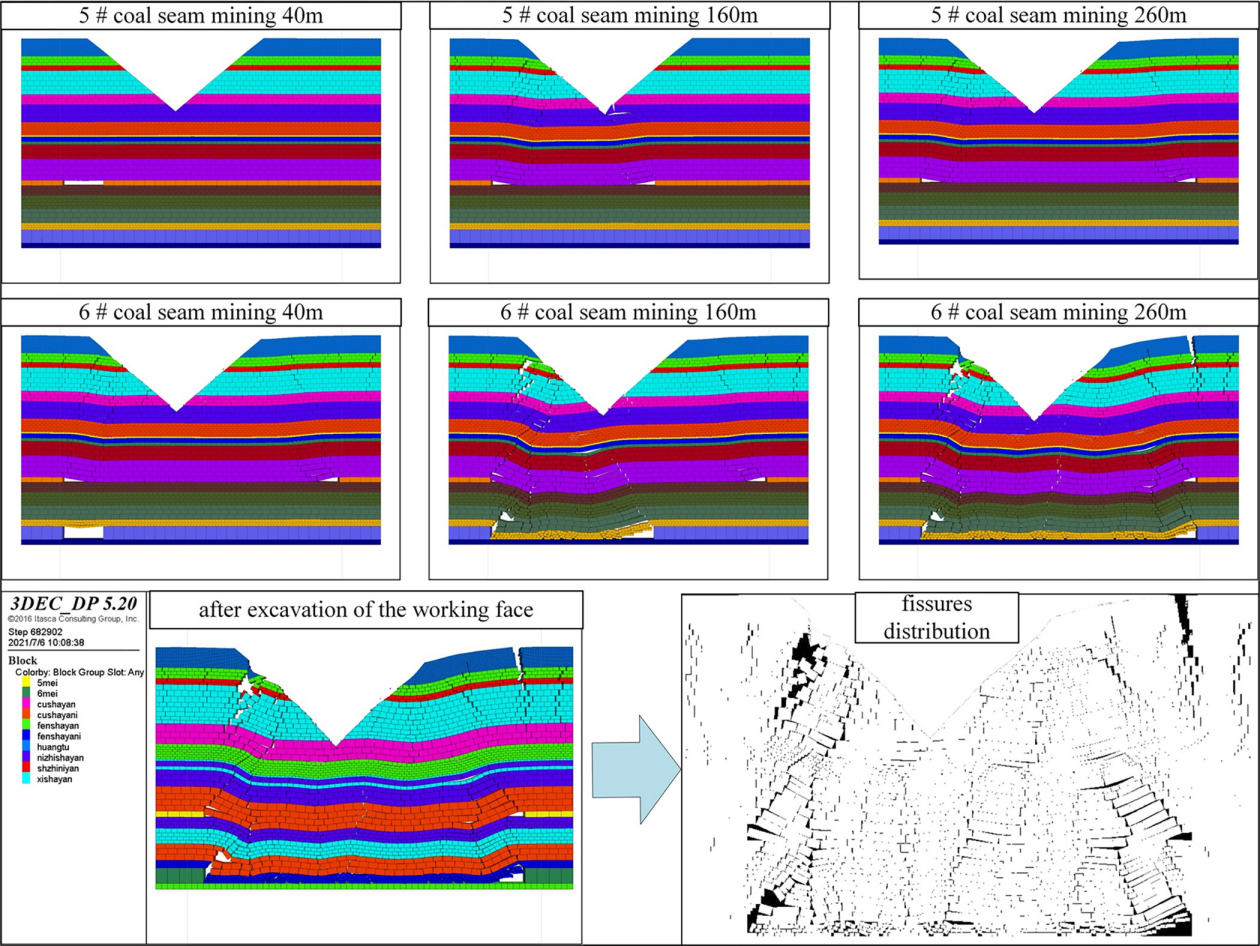
Schematic diagram of model excavation.

In the model excavation process, 40m long protective coal pillars were left on both sides of the model. First, excavate the 5# coal seam, the mining height of the 5# coal seam is 4.6 m, and the step distance of each excavation is 10 m, and the mining length is 260 m. Then excavate the 6# coal seam, the mining height of the 6# coal seam is 13 m, the excavation step is 10 m, and the mining length is 260 m. As shown in Fig 6.

### 4.2. Calculation of fractal dimension of overburden fissures

**Fig 7:**
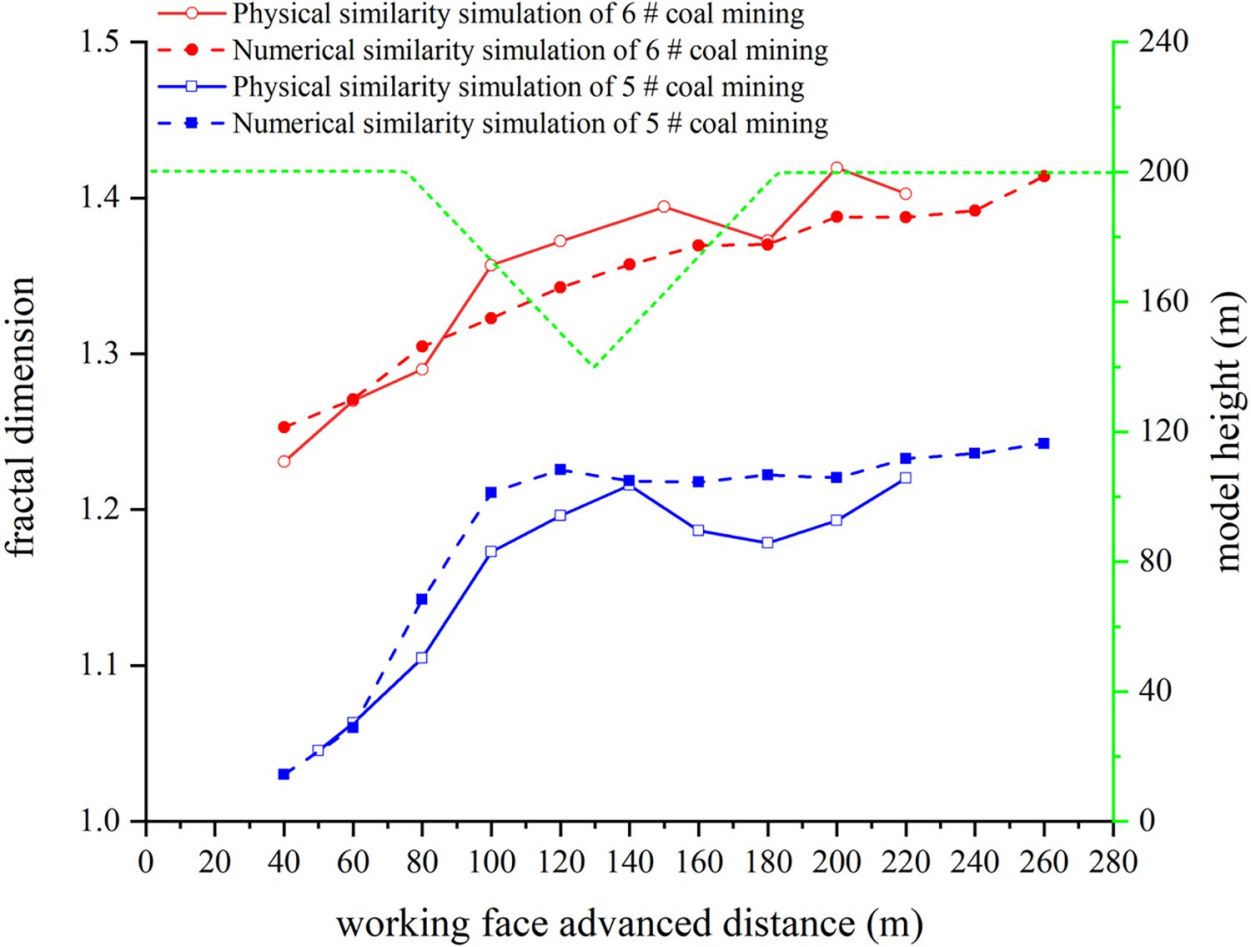
Line chart of fractal dimension.

After the coal seam excavation of the model is completed and the fractal dimension of the overburden fissures with different advancing distances is calculated, as shown in Table 5. Draw a line graph according to the fractal dimension calculation results of the numerical similarity simulation and the physical similarity simulation, as shown in Fig 7.

**Table 5:** Numerical similarity simulation of fractal dimension of overburden fissures after coal mining.

Advancing Distance (m)	fractal dimension	
	5# coal seam mining	6# coal seam mining
40	1.0302	1.2529
60	1.0602	1.2708
80	1.1423	1.3047
100	1.2109	1.3227
120	1.2255	1.3426
140	1.2184	1.3572
160	1.2178	1.3696
180	1.2223	1.3701
200	1.2205	1.3880
220	1.2328	1.3875
240	1.2360	1.3919
260	1.2424	1.4140

It can be seen from Fig 7. In numerical similarity simulations, fine fissures can also be retained because the overburden fissures are easier to identify by the computer. The fractal dimension of the overburden fissures has a smaller range of increase or decrease, and the results of the fractal dimension are more accurate. The variation law of the fractal dimension of overburden fissures in numerical similarity simulation and physical similarity simulation is similar. In the numerical similarity simulation, the change law of the fissures of the overlying rock in the goaf can still be divided into three stages. The three stages are: overburden fissures increase during mining under the left slope, overburden fissures decrease during mining under the right slope, and overburden fissures decrease during mining under the right slope.

### 4.3. Influence of mining stress on fractal dimension

**Fig 8:**
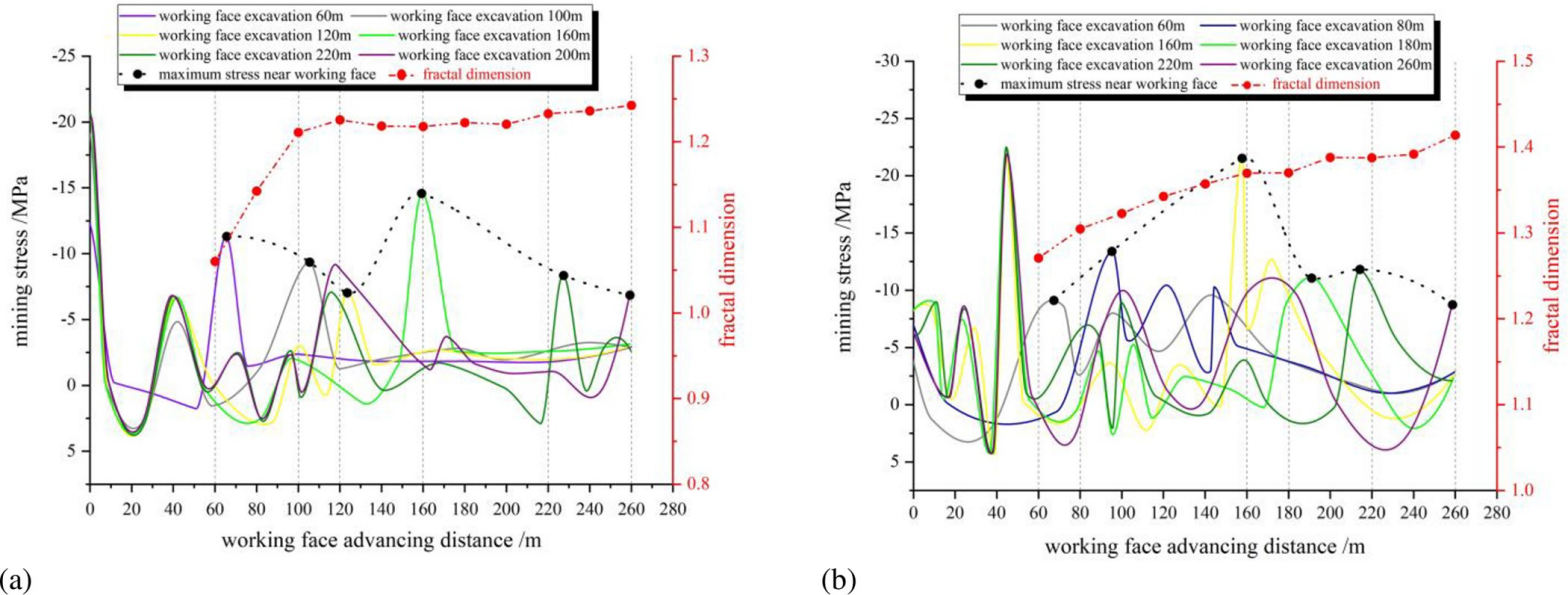
Mining stress and fractal dimension change. (a) 5# coal seam mining; (b) 6# coal seam mining.

The survey lines were set up at the roof positions of the 5# coal seam and the 6# coal seam of the numerical simulation model, and the change of mining stress during the mining process of the working face was monitored, as shown in Fig 8.

Combined with Fig 8, the relationship between mining stress and fractal dimension is analyzed.

#### 4.3.1. 5# coal mining

When the advancing range of the working face is 0~120m, the mining stress on the working face gradually decreases, but the fractal dimension of the overburden fissures gradually increases. When the working face is advanced to 120m, the mining stress on the working face is the smallest, but the fractal dimension of the overburden fissures is the largest.

When the advancing range of the working face is 120~160m, the mining stress on the working face gradually increases, but the fractal dimension of the overburden fissures gradually decreases. When the working face is advanced to 160m, the mining stress on the working face is the largest, but the fractal dimension of the overburden fissures is the smallest.

When the advancing range of the working face is 160~220m, the mining stress on the working face gradually decreases, but the fractal dimension of the overburden fissures gradually increases.

According to the above analysis, the mining stress is negatively correlated with the fractal dimension.

#### 4.3.2. 6# coal mining

During the mining of 6# coal, part of the overlying strata has been destroyed. Under the influence of repeated mining, the instability movement of the overlying rock blocks becomes more complicated, and the relationship between the mining stress and the fractal dimension of the overburden fissures also becomes more complicated.

If one studies the mining of coal seams below the level surface [31], the relationship between stress and fractal dimension of overburden fissures is " The larger mining stress may cause the more complex fissures, and the fractal dimension should be larger ". The relationship between mining stress and fracture fractal dimension becomes more complicated when studying coal seams under the gully surface. The change of mining stress will be affected by the thickness of the surface overlying rock, and also by the movement of the slopes on both sides of the gully. Moreover, the mining stress does not only appear at the working face, but also changes in the goaf. The number of stress fluctuations in the goaf gradually increases with the advancement of the working face. Changes in mining stress at the working face and in the goaf jointly affect the fractal dimension of the overburden fissures.

## 5. Distribution characteristics of overburden fissures in different areas

### 5.1. Partitioning and fractal dimension of overlying rock in goaf

**Fig 9:**
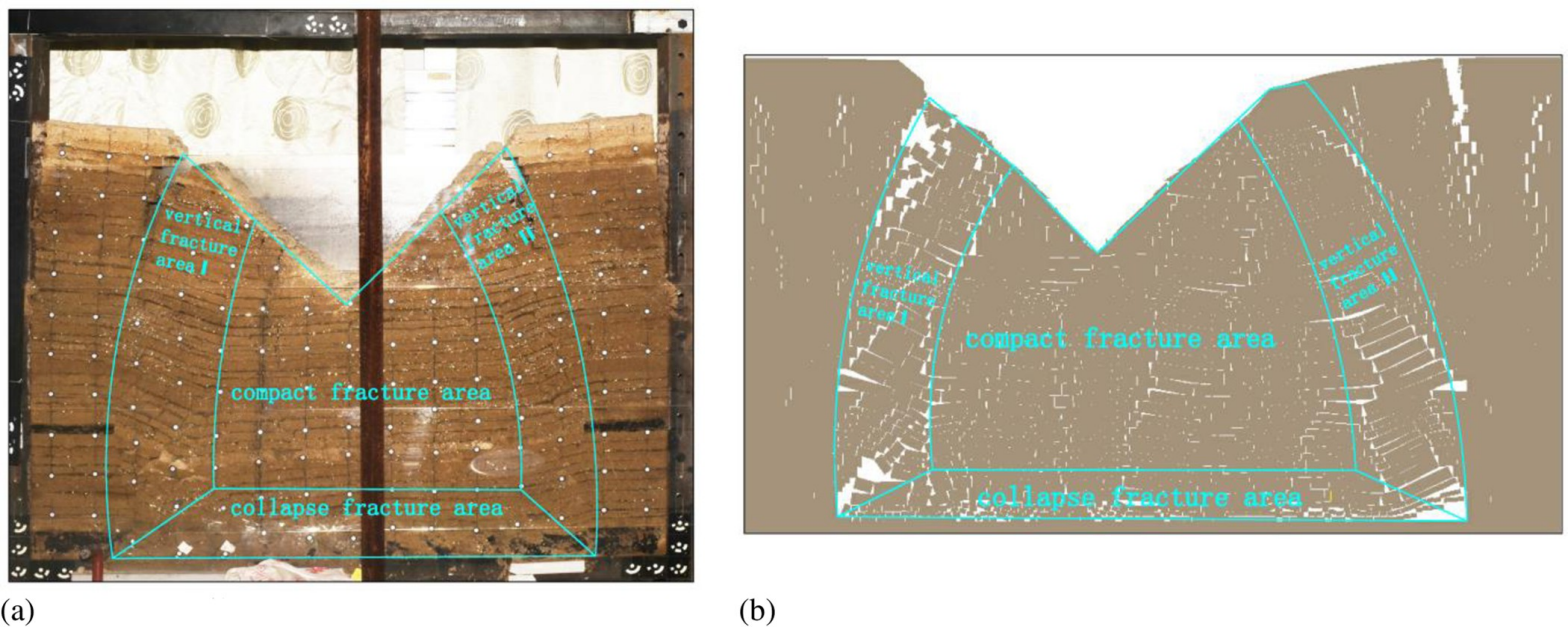
Division of overlying rock. (a) physical Similarity Simulation; (b) numerical Similarity Simulation.

According to the development form of the overburden fissures, the overlying rock in the goaf is divided into three areas, the collapse fissure area, the compaction fissure area, and the vertical fissure area, as shown in Fig 9. Calculate the fractal dimension of each area, as shown in Table 6.

**Table 6:** Fractal dimension of different areas.

area	fractal dimension	
	physical Similarity Simulation	numerical Similarity Simulation
Vertical fissure area I	1.5170	1.7413
Vertical fissure area II	1.4642	1.6833
Collapse fissure area	1.4630	1.6469
Compacted fissure area	1.4724	1.6979

According to Fig 9 and Table 6, the overburden fissures in the vertical fissure areas I and II are more obvious, the penetration is good, and the fractal dimension is relatively large. The overburden fissures in the collapse fissure area are small, dense and inconspicuous, and the fractal dimension is the smallest. The distribution of overburden fissures in the compacted fissure area is relatively regular and the fractal dimension is relatively large.

### 5.2. Distribution characteristics of overburden fissures in different areas

**Fig 10:**
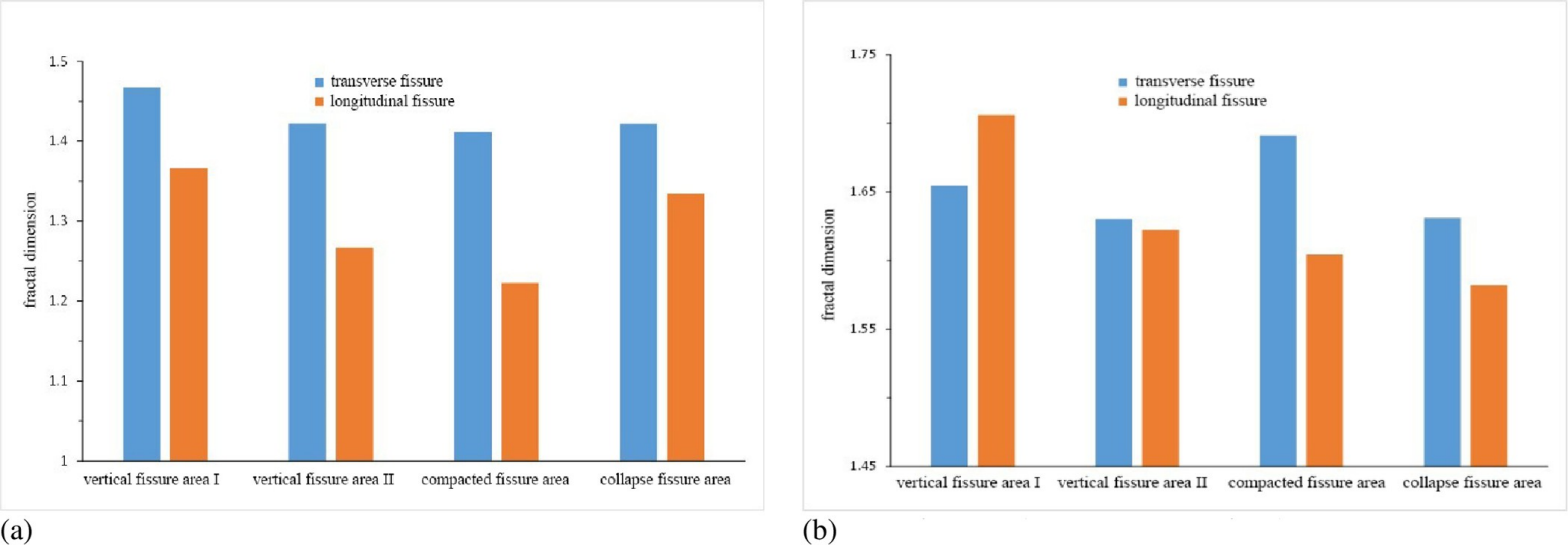
Fractal dimension of transverse and longitudinal fissures in each region. (a) Statistical results of similar simulation; (b) Statistical results of numerical simulation.

According to the different dip angles of the overburden fissures, the fissures in each area are classified. If the dip angle of the fissure is greater than 45°, the fissure is called a longitudinal fissure. If the dip angle of the fracture is less than 45°, the fissure is called a transverse fissure. And calculate the fractal dimension of longitudinal fissures and transverse fissures, as shown in Fig 10.

It can be seen in Fig 10 that the distribution state of the overburden fissures is different in different areas.

#### 5.2.1. Collapse fissure area

The fractal dimensions of the transverse and longitudinal fissures are close to and minimum. This area is located at the bottom of the overlying rock in the goaf. The overlying rocks are most affected by coal seam mining, the overlying rocks are violently unstable, and the collapsed rocks are irregularly deposited in the goaf area, resulting in an irregular distribution of the fissure dip angles. There are many overlying strata in this area, and the self-weight stress is relatively large, some fissures are compacted and closed. Therefore, the overburden fissures in this area are few and are irregularly distributed.

#### 5.2.2. Compaction fissure area

The fractal dimension of the transverse fissures in this area is the largest. Because this area is in the center of the overlying rock in the goaf, the overlying rocks are relatively regular in instability and fracture movement, and the formed fissures are also relatively regular. Affected by the topography of the gully, the overlying rocks on the slopes on both sides of the gully may move toward the center of the gully, and some longitudinal fissures in the overlying rocks are squeezed and disappeared. Therefore, there are many transverse fissures in this area.

#### 5.2.3. Vertical fissure area

The fractal dimensions of transverse and longitudinal fissures in this area are large. This area is distributed on both sides of the overlying rock in the goaf. Affected by the movement of the gully overlying rock, the fissures develop violently and are not easy to close, which is prone to permanent damage to the fissures. There are more longitudinal fissures in this area.

## 6. Results and discussion

Applying fractal theory to the study of the development law of overburden fissures, we find that fractal theory can express the number of overburden fissures in the form of numbers, which is very helpful for the study of overburden fissures.

The development law of overburden fissures in the gully area and the change law of mining stress on the working face are studied. We found that the gully topography has an important influence on the development of overburden fissures, and mining stress has a certain influence on the production and closure of overburden fissures. These conclusions can be applied to actual mining engineering to predict the location of overburden fissures, or to prevent mining stress hazards at working faces.

According to the division of the overlying rock in the goaf, the fissures in the overlying rock in the center of the gully are easily compacted, and the fissures in the overlying rock on the slopes on both sides of the gully are easily opened. That is to say, the overburden fissures are mainly distributed in the slopes on both sides of the overlying rock of the gully, rather than in the overlying rock in the center of the gully. This is useful for remediating ravine surfaces damaged by mining impacts. For example, when filling and repairing the fissures on the surface of the gully, we should focus on repairing the surface fissures on the slopes on both sides.

Finally, this experimental method described in the article still has some shortcomings. For example, in the simulation of physical similarity, there are some errors in the calculation of the fractal dimension. The application of numerical similarity simulation can well solve the above problems. Therefore, in the future research, we should apply more numerical simulations to study some complex problems.

## 7. Conclusions

In this paper, the research method combining similarity simulation and fractal theory is used to analyze the fissures in the overlying rock, and study the development law of the fissures in the overlying rock affected by coal mining in the loess gully area. According to the changing law of mining stress on the roof of coal seam, the influence of mining stress on the development of overlying fissures is analyzed. And based on the distribution characteristics of overburden fissures, the overlying rock in the goaf is divided. Got the following conclusion:

Based on the influence of gully topography on the development of overburden fissures, the development law of overburden fissures in the loess gully area can be divided into three stages; that is, overburden fissures increase during mining under the left slope, the overburden fissures decrease during mining under the right slope, and the overburden fissures decrease during mining under the right slope.

According to the variation law of mining stress, the maximum stress at the working face varies with the thickness of the overlying rock and the number of mining stress fluctuations in the goaf increases with the advancement of the working face. The combined action of the mining stress at the working face and the fluctuation of the stress in the goaf affects the development law of the overburden fissures.

Based on the distribution characteristics of the overburden fissures, the overlying rock in the goaf can be divided into three regions: collapse fissure area, compaction fissure area, and vertical fissure area. Overburden fissures in collapse fissures are few and are irregularly distributed. There are many transverse fissures in the compaction fissure area. There are more longitudinal fissures in the vertical fissure area and the fissures develop violently and are not easy to close.
